# Best Practices for Point of Care Ultrasound: An Interdisciplinary Expert Consensus

**DOI:** 10.24908/pocus.v9i1.17240

**Published:** 2024-04-22

**Authors:** Brandon Oto, Robert Baeten, Leon Chen, Puja Dalal, Ria Dancel, Steven Fox, Carl William Lange IV, Cameron Baston, Paul Bornemann, Siddharth Dugar, Andrew Goldsmith, Meghan Kelly Herbst, James N Kirkpatrick, Abhilash Koratala, Michael J Lanspa, Viveta Lobo, Jason T Nomura, Aliaksei Pustavoitau, Mourad H Senussi, Vincent L. Sorrell, Frances Mae West, Aarti Sarwal

**Affiliations:** 1 Bridgeport Hospital, Yale New Haven Health Bridgeport, CT USA; 2 Piedmont Heart Institute Atlanta , GA USA; 3 Memorial Sloan Kettering Cancer Center New York, NY USA; 4 Novant Health Cornelius, NC USA; 5 University of North Carolina at Chapel Hill Chapel Hill, NC USA; 6 University of Alabama at Birmingham Birmingham, AL USA; 7 Phelps Health Rolla, MO USA; 8 Penn Medicine Philadelphia, PA USA; 9 Lexington Medical Center West Columbia, SC USA; 10 Cleveland Clinic Cleveland, OH USA; 11 Brigham and Women's Hospital Boston, MA USA; 12 UConn Health Farmington, CT USA; 13 University of Washington Seattle, WA USA; 14 Medical College of Wisconsin Milwaukee , WI USA; 15 Intermountain Medical Center and the University of Utah Murray , UT USA; 16 Stanford University School of Medicine Stanford, CA USA; 17 Christiana Care Health System Newark , DE USA; 18 Johns Hopkins University Baltimore , MD USA; 19 Texas Heart Institute, Baylor College of Medicine Houston, TX USA; 20 Gill Heart and Vascular Institute, University of Kentucky Lexington, KY USA; 21 Thomas Jefferson University Hospital Philadelphia, PA USA; 22 Wake Forest University School of Medicine Winston-Salem, NC USA

**Keywords:** POCUS, Guidelines, Consensus, Best Practice, Sonography, Clinical Ultrasound, Emergency ultrasound

## Abstract

Despite the growing use of point of care ultrasound (POCUS) in contemporary medical practice and the existence of clinical guidelines addressing its specific applications, there remains a lack of standardization and agreement on optimal practices for several areas of POCUS use. The Society of Point of Care Ultrasound (SPOCUS) formed a working group in 2022 to establish a set of recommended best practices for POCUS, applicable to clinicians regardless of their training, specialty, resource setting, or scope of practice. Using a three-round modified Delphi process, a multi-disciplinary panel of 22 POCUS experts based in the United States reached consensus on 57 statements in domains including: (1) The definition and clinical role of POCUS; (2) Training pathways; (3) Credentialing; (4) Cleaning and maintenance of POCUS devices; (5) Consent and education; (6) Security, storage, and sharing of POCUS studies; (7) Uploading, archiving, and reviewing POCUS studies; and (8) Documenting POCUS studies. The consensus statements are provided here. While not intended to establish a standard of care or supersede more targeted guidelines, this document may serve as a useful baseline to guide clinicians, leaders, and systems considering initiation or enhancement of POCUS programs.

## Introduction

Ultrasound is now widely used by clinicians as a real-time bedside diagnostic and monitoring tool, a practice often denoted as point of care ultrasound (POCUS). By virtue of its portability, relatively low cost, and broad applicability for a variety of clinical indications, POCUS use has grown in a grass-roots pattern; different centers and even individual clinicians have implemented ultrasound using diverse workflows and practice patterns, often in the absence of well-defined standards.

Professional groups have released guidance on POCUS use in the form of guidelines, expert consensus statements, or practice recommendations, particularly from specialties with high levels of POCUS uptake, such as emergency medicine and critical care 1, 2, 3, 4, 5, 6, 7, 8, 9, 10, 11, 12. However, most guidelines have focused on evidence-based recommendations for the specific clinical uses of POCUS common to their specialty setting. Less effort has been made to establish best practices for POCUS as a generalizable imaging modality, as dictated by the intrinsic characteristics of the tool itself rather than its use-cases for certain subsets of users. Moreover, for many practical questions surrounding POCUS administration and workflows, data are limited. Good practices are instead defined by the subjective perception of a POCUS workflow that is efficient, safe, and ethical for clinicians, learners, and patients. Such questions may be best addressed via expert consensus.

## Methods

With the goal of establishing a set of POCUS best practices with broad applicability, the Society of Point of Care Ultrasound (SPOCUS) formed a multi-disciplinary working group in 2022 (BO, RB, LC, PD, RD, SF, CL).

Between November 2022 and May 2023, the working group drafted a preliminary set of statements related to POCUS use, focusing the content in areas of perceived practice variation, common workflow questions, and a review of existing literature and practice guidelines. As the content focused on areas with limited evidence, the supporting literature review was informal and not structured.

After establishing the initial statement set, a larger panel of POCUS experts was recruited via email (Table 1 for brief panel composition; full member details in Appendix A). Acknowledging that best practices may be specific to country of practice, all experts were based in the United States. All were highly experienced in the clinical use of POCUS; the majority were providers of POCUS training and education; and most held administrative positions in POCUS programs. Otherwise, the panel composition was selected to include diversity of both specialty and practice setting. Specialties represented included emergency and prehospital medicine, critical care and pulmonology, internal medicine and pediatrics, family medicine, neurology, cardiology, anesthesiology, and nephrology; their practice settings included both community and academic institutions, as well as both inpatient and outpatient environments. The panel's clinical background included both physicians and non-physicians (nurse practitioners and physician assistants) with a variety of generalized, specialized, and practical training in clinical ultrasound; their areas of expertise spanned echocardiography, lung and abdominal ultrasound, neurosonography, transesophageal echocardiography, and other modalities.

**Table 1:** Expert panel composition

**Anesthesiology and Critical Care**
Aliaksei Pustavoitau, MD, MHS, FCCM
**Emergency Medicine**
Andrew Goldsmith, MD, MBA Meghan Kelly Herbst, MD, FACEP Viveta Lobo, MD, FACEP
**Emergency and Prehospital Medicine**
Carl William Lange, IV, MSBS, EM-CAQ, PA-C
**Emergency and Internal Medicine**
Jason T Nomura, MD, FACEP, FAAEM, FACP, FAHA
**Cardiology**
James N. Kirkpatrick, MD, FASE, FACC Mourad H Senussi, MD, MS Vincent L. Sorrell, MD, FACP (honorary), FACC, FASE, FSCCT, FSCMR
**Critical Care and Pulmonology**
Cameron Baston, MD, MSCE, FACP Steven Fox, MD Frances Mae West, MD, MS, FACP
**Critical Care Medicine**
Robert Baeten, PA-C, FCCP Leon Chen, DNP, AGACNP-BC, FCCP, FAANP, FCCM Siddharth Dugar, MD, FCCM, FASE, FCCP Michael J. Lanspa, MD Brandon Oto, PA-C, FCCM
**Family Medicine**
Paul Bornemann, MD, RMSK, RPVI Puja Dalal, MD, FAAFP
**Internal Medicine and Pediatrics**
Ria Dancel, MD, FACP, SFHM, FAAP
**Nephrology**
Abhilash Koratala, MD, FASN
**Neurology and Neurocritical Care**
Aarti Sarwal, MD, FNCS, FAAN, FCCM, FASN, RPNI
See Appendix A for details on panel member affiliations, training, and background; Two members who did not complete the consensus process are not listed

The statements were offered to the full panel for voting between June 2023 and November 2023. The format was three iterative voting rounds using a modified Delphi format 13, 14, 15, 16, 17. The first round was exploratory, with the primary goal of developing the themes and crafting semi-final statements. The second round attempted to reach consensus on as many statements as possible. A third round was considered optional, with the purpose of finalizing any statements with lingering concerns. For each statement, consensus was sought either to accept (agree with) or reject (disagree with) its content as written.

The survey was performed using a web-based platform (SurveyMonkey), and required panelists to express agreement with each statement on a five-point Likert scale from Strongly Disagree to Strongly Agree. A sixth option, This topic is outside my expertise, was allowed in case a panelist was unfamiliar with a specialized topic. Qualitative feedback was also permitted via free text, and respondents were encouraged to offer input on how a statement could be improved, particularly if they voted to reject it. No live meetings occurred, and direct collaboration between panel members was not facilitated.

After each round of voting, responses were tabulated. Votes for This topic is outside my expertise were omitted from the denominator for that statement. Out of the remainder, a statement was considered a candidate for acceptance if the votes in agreement (Agree + Strongly Agree) were ≥75% of the total; a statement was a candidate for rejection if the votes in disagreement (Disagree + Strongly Disagree) were ≥75% of the total. The consensus thresholds were defined before the start of voting. Accepted or rejected statements were removed from further voting.

Statements not meeting the consensus criteria were either modified in response to feedback, combined with other statements, or dropped if they appeared redundant or unlikely to achieve consensus. Reintroduced statements included a summary of results of the prior round of voting, including both the vote counts and the qualitative feedback, both de-identified. Statements could also be reintroduced despite reaching consensus if the qualitative feedback voiced important concerns or the potential for further improvement.

The consensus statements were compiled and reviewed by the panel for final approval. Panelists were advised that the final document was the product of the majority consensus, and need not reflect their individual opinion in all respects.

## Results

**Figure 1:**
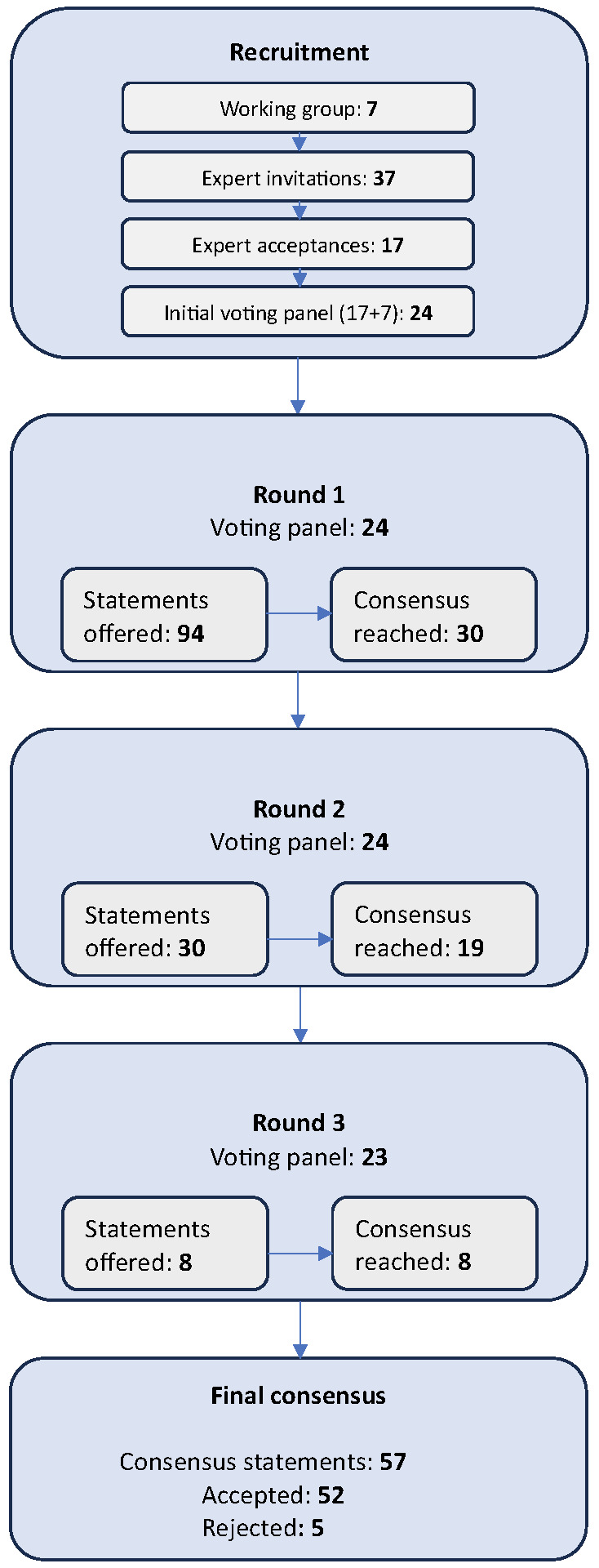
Voting Process.

Panel invitations were extended to 37 experts, and were accepted by 17, creating an initial voting panel of 24 when combined with the seven-person working group (Figure 1).

94 statements were offered in the first voting round, 30 statements in the second, and 8 statements in the third. Of the initial 24 panel members, 23 completed all three voting rounds with 100% responses, while one member dropped out during the second round. A second member completed voting but requested to be omitted from the final consensus statement due to disagreement with one of the accepted statements (see the discussion of Uploading, Archiving, and Review, below). Votes from the two dropout members were included in the data analysis with their permission.

After the three rounds, 57 statements achieved consensus by the panel, with 52 statements accepted and 5 rejected. The consensus statements are shown in Tables 2–12. (Full tabulation of voting results can be found in Appendix B.)

### Definitions

These statements (Table 2) define the terminology used in later statements.

They define a practical definition of POCUS (#1), establish a distinction between POCUS studies performed for clinical purposes and those performed solely for training (#2), and also establish that some studies are non-invasive while others are more invasive in nature (#3); these distinctions are pertinent in later sections (see Credentialing and Consent and Education). The concept of trainees was introduced (#4) separately; this separate distinction allowed study types to be labeled as educational irrespective of the individual performing them, since learners and fully-trained practitioners might perform both clinically-indicated and educational scans. The label “learner” was selected for trainees to be agnostic as to clinical level, since clinicians may learn POCUS at any stage of their training.

These statements were generally uncontroversial, although panelists highlighted that the concept of an “educational” examination (while widely used by both POCUS learners and educators) is relatively unique to POCUS and largely not found in other imaging domains. They also cautioned that these simplified definitions of “invasiveness” may miss distinctions relevant for some applications (see Cleaning and Maintenance).

**Table 2:** Accepted statements (Definitions)

**#**	**Accepted statement**	**Accepted by**
**1**	**Point of care ultrasound (POCUS):** Ultrasound examination performed at the point of care, interpreted and integrated into the clinical context by the treating team. POCUS typically differs from non-POCUS ultrasound by its more targeted scope focused on answering specific clinical questions, and by its real-time acquisition—in most cases with simultaneous interpretation—by the clinical team. POCUS may also be used to identify the need for procedures or interventions, assist in their execution, and confirm their success or identify resulting complications.	91.6%
**2**	**Clinically-indicated study:** A POCUS study performed with the intent of assisting clinical decision-making, such as diagnosis, prognostication, or treatment. Ultrasound used for procedural guidance is included in this category. **Educational study:** A POCUS study performed primarily for education of the self or others, such as practice in image acquisition or demonstration for learners, rather than for a specific clinical indication.	83.3%
**3**	**Non-invasive study**: POCUS examination using surface probes. **Invasive study**: POCUS examination using invasive transducers, such as transesophageal, transvaginal, or transrectal studies.	83.3%
**4**	**Learner**: A clinician not qualified to independently perform and interpret a given modality, technique, or clinical application of POCUS.	83.3%

### Role of POCUS

The single statement in this section (Table 3) establishes the distinction between POCUS and other ultrasound techniques. Initial versions emphasized that POCUS exams are usually more focused than other ultrasound studies and do not serve to replace them. However, a significant number of respondents noted this is not always true; in certain contexts, POCUS exams (particularly when supported by an adequate infrastructure; see Documentation as well as Study uploading, archival, and review) may be sufficient to preclude the need for other testing. Many respondents also highlighted the repeatability of POCUS, which enables it to serve a greater role in monitoring than other ultrasound tests.

**Table 3:** Accepted statements (Role of POCUS)

**#**	**Accepted statement**	**Accepted by**
**5**	Although their diagnostic roles may overlap, POCUS studies are distinct from imaging studies performed through other workflows, including ultrasound examinations of the same anatomic region. POCUS studies tend to yield more immediate data than other studies, and are more easily repeated to assess for changes over time, but in most cases are not as detailed and comprehensive; therefore, depending on the clinical context, a successful POCUS examination may or may not replace the need for other imaging.	87.5%

### Training pathways

These statements (Table 4) address the means by which clinicians, either during or after their foundational training, can develop competence using POCUS. This section was limited by generalizability (#6), as POCUS training is often heavily molded by its context, such as when occurring during undergraduate education, initial clinical training, residency programs, on-job training, or via self-directed learning 18, 19, 20, 21, 22.

Given these limitations, the panel limited their recommendations to broad themes, deferring to other appropriate guidelines to address specific groups (#8–13). The role of informal or self-directed learning in POCUS was controversial (#7). Here, many of the panel acknowledged its prevalence in POCUS, but urged caution given its pitfalls, such as the potential for unrecognized errors; the panel emphasized the importance of adequate quality assurance processes.

Significant debate also occurred around the concept of “monitored usage” (#13), a phase of training which was felt to overlap significantly with later stages of “hands on practice” and with quality assurance during independent practice. Due to this it was unclear to some of the panel whether such a stage is needed. The final accepted statement emphasizes the flexible applicability of the concept.

**Table 4:** Accepted statements (Training pathways)

**#**	**Accepted statement**	**Accepted by**
**6**	Multiple different approaches to POCUS training exist, often varying by medical background and specialty. Although it may be more feasible for specific populations of learners, it is difficult to describe a generalized model for POCUS training that includes every acceptable pathway to competence and acknowledges the many distinct applications and clinical environments of POCUS use.	87.5%
**7**	For some clinicians and circumstances, informal training or self-directed learning may play a significant role and yield successful results, though expert tutelage and structured practice will generally accelerate skill acquisition. However, verification of skills via a supervisory or quality assurance process is essential for such clinicians, to ensure their knowledge base and practice pattern meets locally accepted standards.	83.3%
**8**	We recommend consulting specialty-specific resources and guidelines for recommendations addressing POCUS education within a given training context.	87.5%
**9**	We suggest the following basic framework, which can be flexibly applied to most situations. In general, initial POCUS training should include three elements: **1. Didactic training** **2. Hands-on practice** **3. Monitored usage**	83.4%
**10**	**Didactic training** will generally include education on the principles of ultrasound physics, probe selection, image optimization, artifact recognition, standard views, and the appearance of normal anatomy and important pathology.	91.7%
**11**	This component of learning is amenable to flexible approaches to instruction, including classroom education, bedside teaching, textbook or online training, “flipped” classroom models, or other formats.	100%
**12**	**Hands-on practice** involves learners performing POCUS under direct supervision. This can initially involve practice using ultrasound models, volunteers, cadavers, or high-fidelity simulators, and eventually transition to practice on live, consenting patients under supervision. Immediate feedback by experts should be provided during this stage to guide both image acquisition and interpretation. As live patients are introduced, the clinical integration of POCUS should be embedded into the training process.	100%
**13**	**Monitored usage** involves clinicians applying POCUS to actual patients in the absence of real-time supervision, but with ongoing monitoring in a more sporadic or asynchronous manner. This is usually performed by expert review of archived images, although expert review in real time (in-person or virtual) is sometimes possible. Monitored usage can be implemented to varying degrees depending on local policy and the needs of the system. It can be utilized as a late stage of training or “transition to practice,” wherein POCUS users are considered competent to acquire and interpret images without direct supervision, but still benefit from expert feedback on technical quality or clinical interpretation. After a clinician acquires independent competence with a given study type, as determined by local standards, clinicians may use POCUS within their established skillset without monitoring. However, institutions may choose to continue expert review of either some or all studies performed by credentialed clinicians for purposes of quality assurance or for ongoing education.	91.3%

### Credentialing

This section (Tables 5 and 6) addresses institutional credentialing and privileges for POCUS-performing clinicians.

There was agreement that any credentialing system should be carefully structured to serve the specific system (#14, #15), and should generally be formed by standards and experts specific to the given specialty and setting (#16). No consensus was reached on the optimal approach in systems without sufficient infrastructure to perform institutional credentialing, such as independent practice or austere environments.

A variety of standardized external certifications or examinations intended to demonstrate POCUS competency now exist, generally fee-based and offered by professional societies or commercial enterprises. For example, the National Board of Echocardiography offers specialty ultrasound certifications (such as the “CCEeXAM” in critical care echocardiography), which are undertaken by some clinicians to demonstrate particular expertise in that domain; the process is associated with fees and is not available to non-physicians 23. The panel rejected such external certifications as a standard requirement of POCUS credentialing (#17), believing them unnecessary if competence can be assessed through local methods. However, the majority did feel that certification programs could sometimes have a role in credentialing when thoughtfully or selectively applied (#18).

The panel noted that ultrasound training occurring during foundational clinical programs might be considered “external,” but was not the intention of this statement. For example, clinical ultrasound is a mandatory element of modern emergency medicine residencies 9, and the American College of Emergency Physicians (ACEP) has issued a policy statement asserting that external certification has no role for residency-trained emergency physicians 24. 

The American Medical Association’s resolution H-230.960 states that hospital requirements for POCUS credentialing for physicians should be guided by standards defined by that specialty, such as the well-established ACEP recommendations (which suggests emergency physicians undertake 25–50 quality-reviewed ultrasound studies in each application prior to independent practice) 9, 25. Mirroring this, the expert panel was unable to recommend any specific criteria applicable across both specialty type and resource setting (#14, #19). It was noted that the challenges to POCUS credentialing mean that supervising it through other pathways, such as internal departmental processes, may sometimes be more effective (#15). 

**Table 5:** Accepted statements (Credentialing)

**#**	**Accepted statement**	**Accepted by**
**14**	Processes for ensuring competence among clinicians performing POCUS should be tailored to the practice environment, including the medical specialty and available resources. Although approaches vary, most should involve some combination of a required volume of experience (including both a minimum total number of examinations performed, and minimum pathology encountered), evaluation by a qualified supervisor, and potentially other forms of evaluation such as didactic testing.	87.5%
**15**	Given the heterogeneity of ultrasound applications, it may be difficult to establish privileges for performing POCUS studies that effectively describe the skillsets involved. Few clinicians have competence with every possible application of POCUS, but attempts to define privileges more narrowly (e.g. echocardiography versus abdominal ultrasound) may still lack sufficient breadth and detail to be meaningful for individual clinicians. In general, local privileging strategies for POCUS should be thoughtfully structured to promote safety without creating arbitrary restrictions on practice. More specific privileging for studies with increased risk, such as invasive studies, is usually appropriate. More granular assessments of POCUS skill (e.g. competence examining specific anatomy or using specific modalities) may sometimes be better performed through other pathways, such as departmental supervisory and mentoring structures.	81.8%
**16**	Local standards and methods for establishing POCUS competence should be determined by experts in the specialty and practice environment in which it is being used; standards appropriate for one clinical setting may not apply in another.	87.5%
**18**	In some local credentialing processes, external courses, certifications, or examinations intended to demonstrate a baseline of POCUS training may play a useful role. However, as a broad approach to verifying competence, such standardized tools should neither be considered mandatory (as they may be superfluous for some clinicians to achieve and demonstrate competence), nor necessarily sufficient (in the absence of other training). Without tailoring for the local environment, we do not recommend the general requirement for clinicians to satisfy external standards before credentialing for POCUS use.	82.4%
**19**	Achieving POCUS competency requires experience with its use. In most cases, a sound credentialing standard should therefore require clinicians to perform a pre-defined number of studies prior to allowing unsupervised practice. However, as skill develops non-linearly and at different rates in different clinicians, it is reasonable to establish this minimum threshold at a relatively low number, and it must be combined with a qualitative evaluation of competency that assesses actual knowledge base and practical skills.	87.5%

**Table 6:** Rejected statements (Credentialing)

**#**	**Rejected statement**	**Rejected by**
**17**	External courses, certifications, or examinations intended to demonstrate a baseline of POCUS training should be a requirement of local credentialing processes.	79.1%

### Cleaning and Maintenance

This section (Table 7) addresses maintenance of POCUS equipment in a safe and functional state.

There was difficulty in reaching consensus on standards of device disinfection, partly driven by inconsistencies in the existing recommendations issued by the Center for Disease Control, the American Institute of Ultrasound in Medicine, and an intersocietal position statement issued jointly by 20+ professional clinical groups 26, 27, 28, 29. Guidelines have inconsistently applied the concept of “intermediate level disinfection” (a category between low-level and high-level disinfection not found in all standards) and offer mixed guidance on the disinfection of transducers used for percutaneous procedures such as vascular access (i.e. low- vs high-level disinfection), as well as whether such processing should occur before or after the procedure. Given the high stakes involved in device disinfection, the panel elected to avoid specific recommendations, instead merely reinforcing the importance of following applicable standards, regulations, and manufacturer recommendations (#20).

To address common clinical pitfalls, the panel did emphasize that transducers should be cleaned between patient encounters (#21), as should other components of POCUS devices that become contaminated (#22). The majority favored protecting probes with non-porous covers prior to percutaneous procedures (#21), in contrast with placing a dressing over the transducer face or using disinfection alone. They recommended limiting extra supplies carried on POCUS machines (#26), a common practice that creates inevitable challenges to decontamination, although they acknowledged this was not always practical. A recommendation was made to consider covering portions of the POCUS device with a protective barrier (#23) during encounters with highly contagious airborne or aerosol particles. This method emerged largely during the COVID-19 pandemic; however, the panel acknowledged this practice is lacking in evidence, is not mandatory, and is not sufficient to replace other decontamination measures. 

Transducers in heavy use may become damaged, such as by cracks or chips in the case material, and often remain in use despite these defects. The panel recommended against such use (#24), primarily due to the increased barriers to adequate disinfection. However, they acknowledged that using such devices might be necessary in some cases, and allowed this if the transducer is covered to protect the defect (#25).

**Table 7:** Accepted statements (Cleaning and Maintenance)

**#**	**Accepted statement**	**Accepted by**
**20**	Procedures for cleaning and maintenance of POCUS devices should generally adhere to manufacturer recommendations, as well as pertinent federal, state, and institutional policies. Consideration should be made of the type of exposure, the level of disinfection required based on clinical application, and manufacturer guidelines for the specific equipment. We suggest the following general recommendations which will apply in most circumstances.	95.8%
**21**	Between patient encounters, non-invasive transducers should be cleaned of gross contaminants, then disinfected using an approved agent. Transducers that may contact non-intact skin or bodily fluids, such as during percutaneous procedures, should first be covered with a transducer cover (sterile or clean as determined by the standards of the procedure); disinfection must be performed regardless of the use of a transducer cover. Transducers that will contact mucous membranes (e.g. during transesophageal or transvaginal studies), enter body cavities or the bloodstream, or be used in surgical procedures should be processed in accordance with local policy.	95.7%
**22**	Secondary components of the ultrasound device with the potential for surface contamination, such as cables, control panels, displays, and storage bins, should be disinfected after each patient encounter.	87.5%
**23**	During patient encounters involving exposure to highly contagious aerosolized droplets or airborne particles, the use of barrier devices (e.g. drapes or covers) to cover portions of the ultrasound device should be considered; this measure does not replace the need for appropriate decontamination, but may serve as an adjunct by limiting exposure of secondary device surfaces.	91.3%
**24**	Transducers with visible cracks or penetrating surface defects cannot be adequately cleaned, and should not be used for patient examination until repaired or replaced.	91.3%
**25**	Such damaged transducers may be used in exigent or resource-limited circumstances if completely covered by a non-porous transducer cover.	85.0%
**26**	Spare supplies stored on POCUS machines, such as gloves, catheters, and containers of gel may become contaminated during patient encounters. Even when individually packaged, their exterior surfaces are difficult or unlikely to be disinfected between patients. While sometimes unavoidable, the storage of supplies on POCUS devices should be limited when possible, and care should be taken to avoid their contamination. Disposables such as single-use gel packets should be discarded after each patient encounter.	91.6%

### Consent and Education

This section (Table 8) focuses on the role of patient consent for POCUS studies, and the overlapping topic of POCUS performed for practice or education.

The panel agreed that POCUS studies may be observed by learners if patients allow (#27), and that learners may perform non-invasive studies if adequately supervised (#28). Despite this agreement, the panel was highly divided on the role of learners in invasive studies. While accepting that appropriately-supervised learners may be involved in performing invasive studies, such as transesophageal or transvaginal ultrasound, the majority felt that patient consent was needed for this (#29).

Some respondents felt that non-indicated invasive studies should never be performed for educational reasons alone, but the majority acknowledged that rare exceptions might exist, such as certain didactic situations (e.g. models volunteering for training programs). They agreed that explicitly documented consent must be obtained for these unusual situations (#29).

The panel was unable to agree on the role of consent for non-invasive educational studies, agreeing that they should not be performed on actively dissenting individuals, but acknowledging that non-invasive exams are commonly performed on comatose or sedated patients for practice or teaching. Some experts raised doubts about the ethical basis of this practice, while others wondered whether its acceptability depended on whether it fell under umbrella consents for educational activities in teaching facilities. In the end, no consensus could be reached. Because of this, it was suggested that the topic could be more fully explored in another venue, such as an ethics panel that included patient representatives.

There was clear agreement that learners should not act on POCUS findings unless their findings were first reviewed by an expert (#32). Indeed, the majority felt that learners should generally not perform educational studies without either real-time expert supervision, the availability of timely expert review, or other imaging available to correlate their findings (#30).

**Table 8:** Accepted statements (Consent and Education)

**#**	**Accepted statement**	**Accepted by**
**27**	Learners may observe the performance of any POCUS study if the patient or surrogate decision-maker does not object.	100%
**28**	Non-invasive studies, either clinically-indicated or educational, may be performed by learners if appropriately supervised.	100%
**29**	Consent should be obtained for learner participation in clinically-indicated invasive studies. Outside of uncommon situations, such as educational models, invasive studies should not be performed purely for educational purposes. Explicit consent must be obtained and documented for invasive educational studies.	82.6%
**30**	In order to avoid discovering ultrasound findings of unclear significance, educational studies should not be performed by learners without either: 1. The presence or immediate availability of an expert to validate their findings in real time 2. Other definitive imaging already depicting the area of interest or 3. A quality assurance process that includes archival and timely expert review of all educational studies.	83.3%
**31**	If pathology is identified during an educational study which was not already diagnosed by other means (e.g. prior imaging), expert guidance should be obtained, the primary clinical team informed, and confirmatory imaging considered.	100%
**32**	Learners performing POCUS prior to achieving independent competence should not incorporate their findings into medical decision-making prior to expert review.	87%

### Security, storage, and sharing

This section (Tables 9 and 10) addresses how POCUS exams are stored, as well as non-clinical sharing of images.

The panel acknowledged that POCUS images or clips are often reused outside the clinical context, such as for classroom education or even posts on social media (#33) 30. However, they emphasized that such reproduction should only occur after scrupulous eradication of patient identifiers (#35), which in some cases might include redacting the date of acquisition (#36). When especially rare pathology is depicted, deidentification might require even greater efforts at obscuring the source (#37). They also highlighted the importance of using professional language (#38) when POCUS cases were discussed in public media 31, 32, 33, 34, 35. Despite these cautions, they rejected the idea that properly-anonymized clinical POCUS images should never be reproduced or discussed in public, or that such use always requires patient consent (#34).

The panel noted that studies saved on local device storage present a potential for privacy violations. They suggested that this risk can be mitigated through various methods, including password protection, limiting physical access to devices, or periodic deletion of stored studies (#39). Given the practical and logistical barriers to each of these methods, respondents were reluctant to mandate any of them (for example, recognizing that POCUS machines in busy clinical areas cannot always be stored in locked rooms), but did recommend considering periodic deletion of patient information when device access could not be completely restricted.

Particular caution was urged when personal POCUS devices are independently purchased and used by clinicians (#40). The panel universally agreed that patient information should not be stored on such devices unless they meet the same regulatory and privacy standards as other clinical devices. They also noted that such personal devices often interface with phones or tablets, or upload to cloud-based storage, and each step of this process must also adhere to the same security standards.

**Table 9:** Accepted statements (Security, Storage, and Sharing)

**#**	**Accepted statement**	**Accepted by**
**33**	Clips or images from POCUS studies, whether clinically indicated or acquired for training purposes, are frequently reproduced for teaching. This may include classroom use, lectures in a clinical setting, or digital reproduction on websites, podcasts, or social media.	86.9%
**35**	Images should not be stored, reproduced, shared, or utilized in any form outside the secured medical infrastructure without de-identification. This process should include redaction of the patient’s name, identifier numbers, and date of birth.	87.0%
**36**	Combined with other clinical context, time stamps that include the date of image acquisition may be sufficient to identify the source patient, and should be redacted along with other patient identifiers.	82.6%
**37**	Depictions of rare pathology require additional efforts at obfuscation to prevent identification of the source patient. This may include “fictionalizing” the clinical context not directly relevant to the teaching point, such as gender, age, or secondary clinical features. It may also include delaying usage to establish temporal distance between the case and the reproduction.	95.6%
**38**	When reproduced in public forums such as social media, POCUS cases may be viewed by the public, and should be described using respectful and professional language. Caution should be exercised when clinicians depict cases using humorous or glamorizing language, and derogatory commentary should never be used.	87.0%
**39**	POCUS studies associated with patient identifiers may be retained in local storage on the device, but represent protected healthcare information, and measures must therefore be taken to protect their security. At minimum, this should include limiting physical access to the device, e.g. in locked units or storage areas. In some cases, particularly when physical access cannot be completely restricted (e.g. if patients or visitors may have access), password protection of the device is recommended. Periodically deleting unneeded archives from device storage should be considered as an adjunct to these measures. In settings where neither physical access nor password protection can be adequately achieved, a policy of deleting patient information following each use (after any appropriate archival has been performed) should be considered. We do not recommend long-term archival on local devices without a minimum of password protection.	92.2%
**40**	Personal POCUS devices maintained outside the medical infrastructure may be especially vulnerable to privacy violations. Patient identifiers should not be stored on such devices if they have not been secured in a manner that satisfies federal, state, and local security standards for protected health information, such as password protection and/or the ability to remotely erase stored images in the event of loss or theft. This standard also applies to other devices that may interface with and store footage from portable POCUS transducers, such as mobile phones or portable computers. Remote file storage (i.e. uploading to cloud-based databases) should not occur unless it meets the same standards, which may also apply to the process of electronic transmission, the storage method and permissible usage of the stored files by the parent company, and the security of other downstream devices that may access the stored files after uploading.	100%

**Table 10:** Rejected statements (Security, Storage, and Sharing)

**#**	**Rejected statement**	**Rejected by**
**34**	Regardless of de-identification, POCUS studies obtained during patient care should never be reproduced in public forums (such as the internet or social media) without patient consent.	79.2%

### Uploading, Archival, and Review

This section (Tables 11 and 12) addresses how and when POCUS exams should be saved and reviewed by others.

The panel overwhelmingly agreed that all clinical groups should pursue the infrastructure (e.g. necessary hardware, software, and support) to allow POCUS studies to be saved to the medical record (#42). A method of archival that is readily accessible, such as uploading to a digital radiology system, was heavily preferred (#44); however, in the absence of such infrastructure, it was considered acceptable to save studies using other means, such as printed hardcopies or local storage on the device (#43).

This topic was controversial. In the end, the panel fell just short of mandating that archival must occur universally, allowing the caveat “when resources permit” for niche circumstances or austere environments. They rejected the statement that systems without archival capability should simply not perform POCUS (#41), but repeatedly emphasized that some form of archival is always desirable. (The single expert who dropped out of the consensus process after completion of voting did so due to declining to endorse a recommendation that did not mandate study archival).

The panel agreed that educational studies should generally be saved in some form (#45), but should not be entered into the general medical record; some respondents felt this might be acceptable if educational studies were clearly flagged or labeled as being non-clinical in nature, but the majority believed this created undesirable confusion in the medical record (#46). They agreed that learners should have all studies reviewed by experts in that type of exam (#47, #49), even if this requires informal methods (such as handheld videos of the device display), as long as such methods adhere to privacy standards (#48).

Unlike for educational studies, the panel had more difficulty agreeing on a requirement for expert review of studies performed by credentialed clinicians. Nearly all of the respondents supported a process of review and quality assurance, but there was little consensus on how universally this should occur. Some believed that an expert review process is mandatory and should be achievable in all systems even if it requires flexible approaches. Others felt that exceptions might exist in low-infrastructure settings. In the end, a consensus majority recommended expert review to whatever extent possible, but accepted that systems lacking such resources should not necessarily forbid POCUS use (#50).

The panel was unable to reach consensus on whether expert review constituted a billable clinical service. They felt this was too dependent on regulatory considerations and individual variables.

**Table 11:** Accepted statements (Uploading, Archival, and Review)

**#**	**Accepted statement**	**Accepted by**
**42**	When resources permit, systems should develop infrastructures for the uploading, archival, and shared viewing of POCUS images.	95.7%
**43**	In systems lacking the infrastructure for image uploading, local approaches to image storage should be considered, such as long-term storage on the ultrasound device, archival on local hard drives, or retaining printed hardcopies, although these methods impede the ability of other clinicians to review the findings. Any storage method must offer sufficient privacy and security for storage of healthcare information.	91.3%
**44**	When achievable, uploaded POCUS studies should be accessible by the entire treatment team.	95.6%
**45**	Training, credentialing, and quality assurance are best served when educational studies are saved for review. Depending on local infrastructure, this can be served by uploading them to a separate system which is dedicated to educational imaging and distinct from the clinical record, or by other archival systems such as local storage (e.g. on local discs or plug-in devices). Any method of archival must adhere to local standards of privacy and security.	91.3%
**46**	Educational studies should not be uploaded to imaging archives intended for patient care.	82.6%
**47**	Learners performing POCUS prior to achieving independent competence should have all studies reviewed by an expert.	86.9%
**48**	If review of a study is warranted in a system without the capacity for formal image uploading, it may be achieved using ad hoc methods (such as digitally sharing photographs of device screens), as long as such methods conform to local standards of privacy and security.	78.2%
**49**	Image review should be performed by experts in that type of study.	86.9%
**50**	Whenever resources permit, systems should develop infrastructures and workflows that involve expert review of POCUS studies. Although universal review of all studies may be ideal, review of a selected portion is acceptable, with the fraction determined locally. A process of review is always preferable to no review, and should be established whenever possible. However, in low-resource environments where expert review is not feasible, POCUS use by qualified clinicians should not necessarily be prohibited.	87.0%

**Table 12:** Rejected statements (Uploading, Archival, and Review)

**#**	**Rejected statement**	**Rejected by**
**41**	In systems of care lacking an infrastructure for uploading and archival of images, clinicians should not perform POCUS.	82.6%

### Documentation

This section addresses appropriate documentation of POCUS studies (Tables 13 and 14).

There was universal agreement that the acquisition and interpretation of clinically-indicated studies should be documented in the medical record (#51). This should occur even when the interpretation is performed by a separate individual (#55), as may sometimes happen when consultants or remote clinicians are involved in care. Although somewhat receptive to the idea that certain POCUS applications play a role similar to the physical examination, the panel nevertheless rejected the idea that such studies are exempt from requiring documentation (#57).

The majority supported the use of relatively standardized methods of documentation (#53, #56). Notably, the panel also recommended documenting attempts at clinically-indicated POCUS exams even when they were technically inadequate (#54). 

As with archival (see Uploading, archival, and review), the panel rejected the idea that purely educational studies should be documented in the general medical record (#52), preferring that the record of educational and learning POCUS should remain separate from clinical documentation.

**Table 13:** Accepted statements (Documentation)

**#**	**Accepted statement**	**Accepted by**
**51**	The acquisition and interpretation of clinically indicated studies should be documented in the medical record.	95.7%
**53**	POCUS documentation should, at minimum, include a description of the study performed, its indication, the findings, and the resulting clinical impression.	91.2%
**54**	If a clinically indicated study is attempted, but is inadequate to answer the clinical question(s) for technical reasons, the attempt should be documented.	95.6%
**55**	If image interpretation is performed by a clinician who did not perform the study, such as a consultant or telemedicine provider, the interpreting clinician should document their interpretation in the medical record.	100%
**56**	The acquisition and interpretation of POCUS studies is best documented using standardized note templates.	82.6%

**Table 14:** Rejected statements (Documentation)

**#**	**Rejected statement**	**Rejected by**
**52**	The acquisition and interpretation of educational studies should be documented in the medical record.	82.5%
**57**	Some POCUS studies serve a purpose more analogous to physical examination than to radiographic studies. Such POCUS applications need not be formally documented.	82.6%

## Discussion

This consensus statement is the first known attempt to establish a universal foundation of best practices underpinning POCUS implementation.

It benefits from the diversity of the expert panel as well as the rigorous process of consensus. Although no group of experts can represent the perspective of every practicing clinician, our panel included a broad cross-section of medical specialties, including those with less established footing in POCUS, such as family medicine, neurology, and nephrology. It also included representation from non-physician practitioners, such as physician assistants and nurse practitioners, helping to establish a more general consensus than position statements issued by professional groups with narrower constituencies. Overall, it may serve as a step towards reducing the practice variation that currently exists in the domains addressed, bringing the many disparate implementations of POCUS towards a more unified, consistent standard.

The primary limitations of this document derive from its nature. Regardless of the rigor and diversity of the consensus process, it remains merely an expert consensus, not the direct product of robust evidence. Indeed, many of the topics addressed, such as workflows around documentation and image review, likely have no objectively correct answer.

Additionally, while the final consensus statements were accepted or rejected by ≥75% of the panel, only 12% reached 100% agreement in either direction, implying a lack of universal consensus. The final recommendations can therefore be viewed as a majority opinion, but not one free of controversy.

While diverse, the panel also lacked representation from every potential sub-domain of clinical practice—and even when present, minority perspectives (though potentially valid for certain practice settings) may have been overcome and nullified by the majority vote. No members of non-advanced-practice nursing or active military service were included, nor was there any involvement from patient representatives. The inclusion of these groups could have meaningfully broadened the basis of consensus. Finally, the panel was entirely based in the US and the recommendations were targeted to that setting; international differences in practice were not addressed.

By aiming to describe practices relevant to all users of POCUS, this statement is also limited by the constraints of generalizability. More targeted recommendations may be possible for more specific groups, such as those with shared training (e.g. a background of emergency medicine residency or critical care fellowship), similar practice setting (e.g. hospital wards, outpatient clinics, or the operating room), or consistent needs (e.g. procedural guidance for vascular access or prehospital diagnosis of pneumothorax).

Given the limitations described, this document should not be viewed as a normative guideline describing a standard of care. While the statements included were believed to depict reasonable best practices within the current environment of POCUS in the United States, alternative practices may be appropriate for specific settings or in response to specific needs. The local context, as well as more targeted recommendations or data (where available), must be considered.

Further efforts in this space should consider developing the themes addressed in greater detail, with the ultimate goal of establishing a set of universally-accepted practices that are applicable to POCUS users in every environment. Such a standard should be tested for relevance and appropriateness across multiple specialty settings, and some aspects could potentially be expanded to apply to international clinicians. As stringent criteria can easily be created for high-resource centers that would preclude POCUS use in more austere settings, a universal standard might involve a spectrum of recommendations ranging from optimal practice (appropriate in ideal settings) to minimum acceptable standards (below which POCUS should not be performed).

## Patient Consent Statement

No patients are described in this manuscript. 

## Disclosures

AG has received grants from NIH and consulting fees from Butterfly Network and Ultrasight. AK has received research funding from KidneyCure and the American Society of Nephrology. JK is the chair of the American Society of Echocardiography’s Scientific Statement Writing Group on Nomenclature of cardiac POCUS, and a member of the Critical Care Echocardiography Council Leadership Group.

## Supplementary Material

 Appendix A and Appendix B Appendix A.Expert panel affiliations, training, and background; Appendix B. Full voting results of consensus statements.
