# Supplementary material for: Best Practices for Point of Care Ultrasound: An Interdisciplinary Expert Consensus
Source: POCUS J. 2024 Apr 22;9(1):95–108. doi: 10.24908/pocus.v9i1.17240 (PMC11044939; doi:10.24908/pocus.v9i1.17240)
Supplement: Appendix A and Appendix B [file pocusj-09-17240-s001.pdf]

Appendix A: Expert panel affiliations, training, and background

Anesthesiology and Critical Care

Aliaksei Pustavoitau, MD, MHS, FCCM

Department of Anesthesiology and Critical Care Medicine

Johns Hopkins University

Baltimore, MD

- Associate Professor of Anesthesiology and Critical Care Medicine, Johns Hopkins University School of Medicine
- Director, Perioperative Ultrasound, Department of Anesthesiology and Critical Care Medicine, Johns Hopkins University
- Testamur, NBE Special Competency in Critical Care Echocardiography (CCEeXAM)
- Diplomate, NBE Basic Perioperative Transesophageal Echocardiography (PTEeXAM)
- Instructor, Adult Critical Care Ultrasound course, SCCM
- Co-founder, Introduction to Point-of-Care Ultrasound in Life Support and Sepsis course, SCCM
- Co-director, Point-of-Care Ultrasound in Life Support course, New York State Society of Anesthesiologists Postgraduate Assembly
- Past co-chair, Ultrasound Certification Task Force, SCCM
- Co-editor, *Comprehensive Critical Care Ultrasound (1st ed),* SCCM

Emergency Medicine

Andrew Goldsmith, MD, MBA

Department of Emergency Medicine

Brigham and Women's Hospital

Boston, MA

- Division Chief, Emergency Ultrasound, Brigham and Women's and Faulkner Hospital
- Co-Director, Society of Academic Emergency Medicine Ultrasound-Guided Nerve Block Course

Meghan Kelly Herbst, MD, FACEP

Department of Emergency Medicine

UConn Health

Farmington, CT

- Professor of Emergency Medicine, University of Connecticut School of Medicine
- Director, Point-of-Care Ultrasound, Department of Emergency Medicine, UConn Health
- Chair, Ultrasound Section, ACEP
- Question writer, Advanced Emergency Medicine Ultrasonography Focused Practice Designation Exam, ABEM

Viveta Lobo, MD, FACEP

Department of Emergency Medicine

Stanford University

Stanford, CA

- Clinical Associate Professor, Emergency Medicine, Stanford
- Director of POCUS, Department of Emergency Medicine, Stanford
- Co-Director of POCUS, Stanford Hospital & Clinics
- Board member, Clinical Ultrasound Accreditation Program (CUAP), ACEP

Emergency and Prehospital Medicine

Carl William Lange, IV, MSBS, EM-CAQ, PA-C

1. Emergency Medicine

   Phelps Health

   Rolla, MO

2. Prehospital Medicine PA

   North Crawford County Ambulance District

   Cuba, MO

- CEO and Lead Instructor, Practical POCUS

- Committee Member for PAs in EMS, SEMPA
- SEMPA Liaison, Missouri College of Emergency Physicians
- Adjunct Professor, Saint Louis University Physician Assistant Program
- Adjunct Professor, Missouri State University Physician Assistant Program
- Adjunct Professor, Stephens College Physician Assistant Program

Emergency and Internal Medicine

Jason T Nomura, MD, FACEP, FAAEM, FACP, FAHA

Department of Emergency Medicine

Christiana Care Health System

Newark, DE

- Director, Emergency Ultrasound, Thomas Jefferson University
- Advanced Emergency Medicine Ultrasound Fellowship Director, Christiana Care Health System
- Medical Director, Systemwide Point of Care Ultrasound, Thomas Jefferson University
- Associate Professor of Emergency Medicine & Associate Professor of Medicine, Sidney Kimmel Medical College at Thomas Jefferson University
- Focused Practice Designation Advanced Emergency Medicine Ultrasound (FPD-AEMUS), American Board of Emergency Medicine

Cardiology

James N. Kirkpatrick, MD, FASE, FACC

Division of Cardiology and Department of Bioethics and Humanities

University of Washington

Seattle, WA

- Professor of Medicine, Department of Cardiology, University of Washington
- Professor of Bioethics and Humanities, University of Washington
- Section Chief, Cardiac Imaging, University of Washington Medical Center
- Director, Echocardiography Laboratories, University of Washington Medical Center
- Member, Critical Care Echocardiography Council, ASE
- Chair, ASE Foundation
- Diplomate, NBE Examination of Special Competence in Adult Echocardiography (ASCeXAM)
- Writing Committee, NBE Examination of Special Competence in Adult Echocardiography (ASCeXAM)
- Chair, ASE Guideline: Recommendations for Echocardiography Laboratories Participating in Cardiac Point of Care Cardiac Ultrasound (POCUS) and Critical Care Echocardiography Training: Report from the American Society of Echocardiography
- Chair, ASE Writing Group: Recommendations for Cardiac Point-of-Care Ultrasound Nomenclature

Mourad H Senussi, MD

Cardiology & Critical Care Medicine

Baylor College of Medicine

Texas Heart Institute

Houston, TX

- Assistant Professor, Baylor College of Medicine
- Director, Cardiac Intensive Care Unit, Baylor St. Luke's Medical Center
- Diplomate, NBE Examination of Special Competence in Adult Echocardiography (ASCeXAM)
- Diplomate, NBE Special Competency in Critical Care Echocardiography (CCEeXAM)

Vincent L. Sorrell, MD, FACP (honorary), FACC, FASE, FSCCT, FSCMR

Division of Cardiovascular Medicine

University of Kentucky

Lexington, KY

- Anthony N. DeMaria Professor of Medicine, University of Kentucky
- Chief, Division of Cardiovascular Medicine, University of Kentucky
- Acting Director, Gill Heart & Vascular Institute
- Level III echocardiographer, ACC
- Editor-in-Chief, CASE (Cardiovascular Imaging Case Reports)
- Co-director, Echo Florida, ASE

- Inaugural faculty, Steering Committee, NBE Critical Care Echocardiography Boards Review Course
- Member, Association of University Cardiologists
- Recipient, Richard Popp Excellence in Teaching Award, ASE
- Past member, Board of Directors, ASE
- Writing group member (POCUS nomenclature and standardized reporting), ASE
- Writer, Echocardiography Self-Assessment Program (EchoSAP), ACC
- Writer, Cardiac Catheterization and Interventional Cardiology Self-Assessment Program (CathSAP), ACC

Critical Care Medicine

Robert Baeten, PA-C, FCCP

Cardiac Critical Care Service

Piedmont Heart Institute

Atlanta, GA

- Clinical Assistant Professor, Mercer University Physician Assistant Program
- Director, POCUS Curriculum, Mercer University Physician Assistant Program
- Founder and course director, POCUS for the Acute Care Provider, Mercer University Physician Assistant Program
- Former faculty, Post Graduate PA Residency in Cardiac Critical Care
- Co-creator, Focused Cardiac Ultrasound credentialing and QI workflow, Piedmont Hospital Atlanta

- Former member, Adult Critical Care Ultrasound Committee, SCCM

Leon Chen, DNP, AGACNP-BC, FCCP, FAANP, FCCM

Department of Anesthesiology and Critical Care Medicine

Memorial Sloan Kettering Cancer Center

New York, NY

- Associate Professor of Nursing, Columbia University School of Nursing
- Core POCUS Faculty, DNP/AGACNP/CRNA Programs, Columbia University School of Nursing
- Developed and supervises APP POCUS certification, Intensive Care Unit/Post-Anesthesia Care Unit/Step-Down Units, Memorial Sloan Kettering Cancer Center
- Co-Founder and Course Director, Critical Care Ultrasound Workshop, Memorial Sloan Kettering Cancer Center
- Diplomate, Critical Care Ultrasonography Certificate of Completion, American College of Chest Physicians

Siddharth Dugar, MD, FCCM, FASE, FCCP

Respiratory Institute, Department of Critical Care Medicine

Cleveland Clinic

Cleveland, OH

- Director of Point of Care Ultrasound, Department of Critical Care, Respiratory Institute, Cleveland Clinic

- Diplomate, NBE Special Competency in Critical Care Echocardiography (CCEeXAM)

Michael J. Lanspa, MD

Critical Care Medicine

Intermountain Medical Center and the University of Utah

Murray, UT

- Adjunct Associate Professor, Internal Medicine, University of Utah
- Director, Intermountain Critical Care Echocardiography Service
- Director, Intermountain Critical Care Echocardiography Core Imaging Research Lab
- Co-chair, Scientific Sessions Program Committee, ASE
- Co-chair, Critical Care Echocardiography Board Review course, SCCM
- Co-chair, American Thoracic Society Post Graduate Course: Critical Care Echocardiography and Ultrasonography
- Chair, Utah Thoracic Society Critical Care Echocardiography and Ultrasound Roundtable Summit.
- Course Faculty and Committee Member, Ultrasound Committee, SCCM
- Steering Committee Member, Point of Care Ultrasound Guideline Working Group, SCCM

Brandon Oto, PA-C, FCCM

Medical ICU

Bridgeport Hospital, Yale New Haven Health

Bridgeport, CT

- Clinical Instructor, Department of Medicine, Yale School of Medicine
- Instructor, iScan 2022

Critical Care Medicine and Pulmonology

Cameron Baston, MD, MSCE, FACP

Division of Pulmonary, Allergy and Critical Care

Penn Medicine

Pennsylvania, PA

- Assistant Professor of Clinical Medicine, Penn Medicine
- Associate Program Director, Pulmonary and Critical Care Medicine Fellowship, Penn Medicine
- Director of Point of Care Ultrasound, Department of Medicine, Penn Medicine
- Faculty Associate Director, Penn Health-Tech

Steven Fox, MD

Pulmonology and Critical Care Medicine

University of Alabama at Birmingham

Birmingham, AL

- Assistant Professor of Medicine, University of Alabama at Birmingham School of Medicine

Frances Mae West, MD, MS, FACP

Division of Pulmonary, Allergy & Critical Care Medicine

Thomas Jefferson University Hospital

Philadelphia, PA

- Associate Professor of Medicine, Sidney Kimmel Medical College at Thomas Jefferson University
- Program director, Jefferson Pulmonary Disease & Critical Care Medicine Fellowship
- Program director, Internal Medicine Point-of-Care Ultrasound Fellowship, Thomas Jefferson University Hospital
- Course Director, The Resuscitative TEE Workshop
- Founder and course director, Philadelphia Area Critical Care Ultrasound Program Regional Ultrasound Course
- Testamur, NBE Special Competency in Critical Care Echocardiography (CCEeXAM)
- Member, CCEeXAM Committee, NBE

Family Medicine

Paul Bornemann, MD, RMSK, RPVI

Family Medicine

Lexington Medical Center

West Columbia, SC

- Adjunct Professor of Family Medicine, University of South Carolina School of Medicine
- Director of POCUS Curriculum, Family Medicine and Transitional Year Residency Programs, Lexington Medical Center
- Past director, Primary Care Ultrasound, University of South Carolina School of Medicine
- Founding chair, POCUS Interest Group, AAFP
- Past member, Board of Governors, AIUM
- Author, “Ultrasound for Primary Care”

Puja Dalal, MD, FAAFP

Family Medicine

Novant Health

Cornelius, NC

- Director, Point-of-Care Ultrasound, Novant Health Family Medicine Residency
- Core Faculty, POCUS Certificate Program for Educators, Society for Teachers of Family Medicine
- Graduate, Primary Care POCUS Fellowship, Prisma Health/University of South Carolina

- POCUS Instructor, Society for Teachers of Family Medicine
- Vice Chair, POCUS Member Interest Group, AAFP
- POCUS Instructor, AAFP
- Co-founder and co-chair, POCUS Collaborative, Society for Teachers of Family Medicine
- POCUS Instructor, American College of Physicians
- POCUS Instructor, North Carolina Academy of Family Physicians

Internal Medicine and Pediatrics

Ria Dancel, MD, FACP, SFHM, FAAP

Division of Hospital Medicine

University of North Carolina at Chapel Hill

Chapel Hill, NC

- Professor of Internal Medicine and Pediatrics, University of North Carolina at Chapel Hill
- Director, Medicine Procedure Service, University of North Carolina at Chapel Hill
- Director, Point-of-Care Ultrasound Education, University of North Carolina at Chapel Hill
- Associate Director, Medicine-Pediatrics Residency Program, University of North Carolina at Chapel Hill

Nephrology

Abhilash Koratala, MD, FASN

Division of Nephrology

Medical College of Wisconsin

Milwaukee, WI

- Associate Professor of Medicine, Medical College of Wisconsin
- Director of Clinical Imaging and Social Media, Nephrology, Medical College of Wisconsin
- Testamur, NBE Special Competency in Critical Care Echocardiography (CCEeXAM)
- Member, Guideline writing panel to address the use of ultrasound for adult critically ill and injured patients (focused update 2023), SCCM
- Member, Education Working Group, International Society of Nephrology (ISN)
- Founder, International Alliance for POCUS in Nephrology (IAPN)
- Associate Editor, POCUS Journal
- Chair, POCUS workshop, National Kidney Foundation Spring Clinical Meetings (2022–2024)
- Co-chair, POCUS course, American Society of Nephrology Annual Meeting (2022)
- Member, POCUS certification committee, American Society of Diagnostic and Interventional Nephrology (2020–2022)

Neurology and Neurocritical Care

Aarti Sarwal, MD, FNCS, FAAN, FCCM, FASN, RPNI

Neurology and Neurocritical Care

Atrium Health Wake Forest School of Medicine

Winston-Salem, NC

- Professor of Neurology, Atrium Wake Forest School of Medicine
- Director, Neurovascular Laboratory, Atrium Wake Forest School of Medicine
- Director, Neurovascular Ultrasound Courses (120 CME hours/year), Atrium Wake Forest School of Medicine
- Co-Director, Medical School Ultrasound Curriculum, Atrium Wake Forest School of Medicine
- Assistant Course Director, Point of Care Ultrasound Elective, Atrium Wake Forest School of Medicine
- Member, Ultrasound Committee, SCCM
- Co-Author, COVID-19 update on POCUS applications during the pandemic, ASE [36]
- Co-Author, Expert consensus and recommendations on basic ultrasound skills for intensivists, ESICM [12]
- Member, Guideline writing panel to address the use of ultrasound for adult critically ill and injured patients (focused update 2023), SCCM
- Organizing Chair, 6th World Congress of Society of Ultrasound in Medical Education

- Founding chair, Ultrasound Section, Neurocritical Care Society (2020–2022)
- Co-Director, POCUS workshop, Neurocritical Care Society Annual Meeting (2016, 2017, 2019, 2023)
- Co-Director, POCUS workshop, AIUM (2015, 2016)

POCUS: Point of care ultrasound

AAFP: American Academy of Family Physicians

ABEM: American Board of Emergency Medicine

AIUM: American Institute of Ultrasound in Medicine

ACC: American College of Cardiology

ACEP: American College of Emergency Physicians

ASE: American Society of Echocardiography

NBE: National Board of Echocardiography

SCCM: Society of Critical Care Medicine

SEMPA: Society of Emergency Medicine PAs

Appendix B: Full voting results of consensus statements

Statement #	This topic is outside my expertise (%)	Strongly disagree (%)	Disagree (%)	Neither agree nor disagree (%)	Agree (%)	Strongly agree (%)	Round consensus reached	Total responses
**1**	0 (0)	0 (0)	1 (4.2)	1 (4.2)	9 (37.5)	13 (54.1)	1	24
**2**	0 (0)	0 (0)	2 (8.3)	2 (8.3)	7 (29.2)	13 (54.1)	1	24
**3**	0 (0)	0 (0)	2 (8.3)	2 (8.3)	7 (29.2)	13 (54.1)	1	24
**4**	0 (0)	1 (4.2)	1 (4.2)	2 (8.3)	6 (25.0)	14 (58.3)	2	24
**5**	0 (0)	0 (0)	2 (8.3)	1 (4.2)	9 (37.5)	12 (50.0)	2	24

**6**	0 (0)	0 (0)	1 (4.2)	2 (8.3)	11 (45.8	10 (41.7)	2	24
**7**	0 (0)	0 (0)	0 (0)	4 (16.7)	12 (50.0)	8 (33.3)	2	24
**8**	0 (0)	0 (0)	1 (4.2)	2 (8.3)	10 (41.7)	11 (45.8)	1	24
**9**	0 (0)	0 (0)	0 (0)	4 (16.7)	7 (29.2)	13 (54.2)	1	24
**10**	0 (0)	1 (4.2)	0 (0)	1 (4.2)	9 (37.5)	13 (54.2)	1	24
**11**	0 (0)	0 (0)	0 (0)	0 (0)	12 (50.0)	12 (50.0)	1	24
**12**	1 (4.2)	0 (0)	0 (0)	0 (0)	9 (39.1)	14 (60.9)	2	24
**13**	0 (0)	0 (0)	1 (4.3)	1 (4.3)	9 (39.1)	12 (52.2)	3	23
**14**	0 (0)	0 (0)	0 (0)	3 (12.5)	11 (45.8)	10 (41.7)	2	24
**15**	1 (4.3)	0 (0)	2 (9.1)	2 (9.1)	11 (50.0)	7 (31.8)	3	23
**16**	0 (0)	0 (0)	2 (8.3)	1 (4.2)	9 (37.5)	12 (50.0)	1	24
**17**	0 (0)	5 (20.8)	14 (58.3)	2 (8.3)	2 (8.3)	1 (4.2)	1	24
**18**	0 (0)	1 (4.2)	2 (8.3)	1 (4.2)	10 (41.2)	10 (41.2)	2	24
**19**	0 (0)	1 (4.2)	2 (8.3)	0 (0)	12 (50.0)	9 (37.5)	2	24
**20**	0 (0)	0 (0)	1 (4.2)	0 (0)	12 (50.0)	11 (45.8)	2	24
**21**	0 (0)	0 (0)	1 (4.3)	0 (0)	10 (43.5)	12 (52.2)	3	23
**22**	0 (0)	0 (0)	2 (8.3)	1 (4.2)	14 (58.3)	7 (29.2)	2	24
**23**	0 (0)	0 (0)	2 (8.7)	0 (0)	16 (69.6)	5 (21.7)	3	23
**24**	1 (4.2)	9 (39.1)	1 (4.3)	1 (4.3)	13 (56.5)	8 (34.8)	1	24
**25**	4 (16.7)	0 (0)	0 (0)	3 (15.0)	11 (55.0)	6 (30.0)	1	24
**26**	0 (0)	0 (0)	0 (0)	2 (8.3)	17 (70.8)	5 (20.8)	2	24
**27**	0 (0)	0 (0)	0 (0)	0 (0)	6 (25.0)	18 (75.0)	1	24
**28**	0 (0)	0 (0)	0 (0)	0 (0)	10 (43.5)	13 (56.5)	3	23
**29**	0 (0)	1 (4.3)	3 (13.0)	0 (0)	12 (52.2)	7 (30.4)	3	23
**30**	0 (0)	1 (4.2)	1 (4.2)	2 (8.3)	15 (62.5)	5 (20.8)	2	24
**31**	0 (0)	0 (0)	0 (0)	0 (0)	8 (33.3)	16 (66.6)	1	24
**32**	0 (0)	0 (0)	1 (4.3)	2 (8.7)	12 (52.2)	8 (34.8)	1	23
**33**	0 (0)	0 (0)	0 (0)	3 (13.0)	9 (39.1)	11 (47.8)	1	23
**34**	0 (0)	7 (30.4)	11 (48.8)	1 (4.3)	3 (13.0)	1 (4.3)	3	23
**35**	0 (0)	1 (4.3)	0 (0)	2 (8.7)	2 (8.7)	18 (78.3)	1	23
**36**	0 (0)	0 (0)	1 (4.3)	3 (13.0	9 (39.1	10 (43.5)	1	23
**37**	0 (0)	0 (0)	0 (0)	1 (4.3)	21 (91.3)	1 (4.3)	2	23
**38**	0 (0)	0 (0)	0 (0)	3 (13.0)	10 (43.5)	10 (43.5)	2	23
**39**	0 (0)	0 (0)	1 (4.3)	1 (4.3)	15 (65.2)	6 (26.0)	2	23
**40**	0 (0)	0 (0)	0 (0)	0 (0)	14 (60.9)	9 (39.1)	2	23
**41**	0 (0)	9 (39.1)	10 (43.5)	0 (0)	2 (8.7)	2 (8.7)	1	23
**42**	0 (0)	0 (0)	1 (4.3)	0 (0)	4 (17.4)	18 (78.3)	1	23
**43**	0 (0)	2 (8.7)	0 (0)	0 (0)	16 (69.6)	5 (21.7)	3	23
**44**	0 (0)	0 (0)	0 (0)	1 (4.3)	6 (26.0)	16 (69.6)	2	23
**45**	0 (0)	0 (0)	1 (4.3)	1 (4.3)	14 (60.9)	7 (30.4)	2	23
**46**	0 (0)	0 (0)	2 (8.7)	2 (8.7)	9 (39.1)	10 (43.5)	1	23
**47**	0 (0)	0 (0)	1 (4.3)	2 (8.7)	9 (39.1)	11 (47.8)	1	23
**48**	0 (0)	0 (0)	3 (13.0)	2 (8.7)	11 (47.8)	7 (30.4)	1	23
**49**	0 (0)	1 (4.3)	0 (0)	2 (8.7)	11 (47.8)	9 (39.1)	1	23
**50**	0 (0)	0 (0)	1 (4.3)	2 (8.7)	12 (52.2)	8 (34.8)	2	23
**51**	0 (0)	1 (4.3)	0 (0)	0 (0)	2 (8.7)	20 (87.0)	1	23
**52**	0 (0)	6 (26.0)	13 (56.5)	1 (4.3)	2 (8.7)	1 (4.3)	1	23
**53**	0 (0)	0 (0)	1 (4.3)	1 (4.3)	6 (26.0)	15 (65.2)	1	23
**54**	0 (0)	0 (0)	1 (4.3)	0 (0)	15 (65.2)	7 (30.4)	1	23
**55**	0 (0)	0 (0)	0 (0)	0 (0)	13 (56.5)	10 (43.5)	1	23
**56**	0 (0)	0 (0)	1 (4.3)	3 (13.0)	11 (47.8)	8 (34.8)	1	23
**57**	0 (0)	7 (30.4)	12 (52.2)	3 (13.0)	9 (39.1)	1 (4.3)	1	23
